# Age-Specific Colonoscopic Yield and Symptom-Based Risk Stratification in Symptomatic Adults: A Bicenter Omani Analysis to Inform Early Detection and Screening Strategies for Colorectal Neoplasia

**DOI:** 10.3390/medicina62020374

**Published:** 2026-02-13

**Authors:** Adhari Alzaabi, Hafsa Al Rasbi, Zayana Almaawali, Yaqeen Alyahmadi, Said A. Al-Busafi

**Affiliations:** 1 Department of Human and Clinical Anatomy, College of Medicine and Health Sciences, Sultan Qaboos University, Muscat 123, Oman; 2 College of Medicine and Health Sciences, Sultan Qaboos University, Muscat 123, Oman; 3 Department of Medicine, College of Medicine and Health Sciences, Sultan Qaboos University, Muscat 123, Oman; 4 Medical Research Center, Sultan Qaboos University, Muscat 123, Oman

**Keywords:** colorectal cancer, early-onset colorectal cancer, adenoma detection rate, advanced colorectal neoplasia, colonoscopy, diagnostic yield, screening guidelines, Oman

## Abstract

*Background and Objectives*: Colorectal cancer (CRC) is the most common gastrointestinal malignancy in Oman and among the top three cancers nationally, with an increasing burden of early-onset CRC (EOCRC) diagnosed before age 50. Despite national CRC guidelines, the lack of an organized screening program means most cases are detected symptomatically and at advanced stages. This study evaluated age-related differences in colonoscopic findings and advanced neoplasia risk in symptomatic adults to inform early detection and screening strategy development. *Materials and Methods*: A cross-sectional analysis of 2041 colonoscopies performed at two national tertiary referral centers, Sultan Qaboos University Hospital and Royal Hospital, was conducted (2018–2021). Patients were categorized by age (<50 vs. ≥50 years). Outcomes included adenoma detection rate (ADR), advanced premalignant lesions (APL), colorectal cancer (CRC), and advanced colorectal neoplasia (ACRN; APL and/or CRC). Associations between presenting symptoms and ACRN were analyzed using univariable logistic regression, and diagnostic yield was estimated via number needed to scope (NNS). The cohort was predominantly symptomatic (71.9%), with 15.8% screening and 12.3% surveillance procedures. *Results*: Of 2041 procedures, 742 (36.3%) were in patients <50 years. ADR, APL, CRC, and ACRN were significantly higher in those ≥50 years (14.9%, 7.5%, 5.8%, and 13.3%) than in younger adults (8.5%, 3.2%, 2.7%, and 5.9%; all *p* < 0.01). Among younger adults, rectal bleeding (OR 2.17, 95% CI 1.15–4.08, *p* = 0.026) and abdominal pain (OR 2.14, 95% CI 1.15–3.98, *p* = 0.022) were significantly associated with ACRN. Diagnostic efficiency (NNS) was highest for loss of appetite in both age groups (4.7 in <50 vs. 2.8 in ≥50 years). Despite lower overall rates, a substantial burden of advanced neoplasia was observed in symptomatic adults <50 years (5.9% ACRN, 2.7% CRC). *Conclusions*: This bicenter study demonstrates clear age-related disparities in colorectal neoplasia, with a clinically important burden of advanced disease in symptomatic adults under 50 years. These findings highlight the importance of prompt colonoscopic evaluation for younger adults presenting with alarm symptoms, particularly rectal bleeding and abdominal pain, and provide evidence supporting risk-stratified diagnostic approaches. While age-related differences suggest potential value in earlier screening initiation, our predominantly symptomatic tertiary care cohort cannot directly determine optimal screening age thresholds. Prospective screening trials and cost-effectiveness analyses are needed to establish population-based detection rates and inform evidence-based screening policy development in Oman.

## 1. Introduction

Colorectal cancer (CRC) ranks as the third most common malignancy and the second leading cause of cancer death globally [1]. Despite substantial progress in prevention and screening, CRC incidence remains high in many low- and middle-income regions, including the Middle East, where screening uptake is limited, and public awareness remains low [2]. In Oman, CRC is the most common gastrointestinal malignancy and one of the top three cancers nationally, with age-standardized incidence rates rising from 7.8 per 100,000 in 2005 to 11.2 per 100,000 in 2020 [3,4]. This increasing burden is compounded by late-stage diagnosis: despite national screening guidelines, the absence of an organized population-based screening program means 65–70% of CRC cases are diagnosed at stages III–IV. Early-onset colorectal cancer (EOCRC, diagnosed before age 50) has emerged as a critical concern, with global incidence rising 1–2% annually while rates in older adults decline [5]. In Oman, approximately 30–35% of all CRC cases occur in individuals under 50 years of age, and nearly 20% occur before age 40 [4]. This pattern is consistent across the Middle East region: Saudi Arabia, Kuwait, and the United Arab Emirates report EOCRC proportions exceeding 30%, substantially higher than the 10–15% observed in North America and Europe [4]. The disproportionate EOCRC burden in this region underscores the need for age-group comparison data to inform screening policy. While recent international guidelines now recommend screening from age 45 [6,7], region-specific evidence is needed to determine appropriate screening thresholds for Middle Eastern populations.

Colonoscopy remains the gold standard for CRC prevention, enabling detection and removal of precancerous adenomas. The adenoma detection rate (ADR) is a key quality indicator, with higher ADRs associated with lower interval CRC and mortality [8]. Higher ADRs are independently associated with lower risks of interval CRC and CRC-related mortality [9]. However, data on the age comparison of colonoscopic findings and neoplasia yield in Oman are scarce.

This study, therefore, aimed to compare colonoscopy indications, neoplastic findings, and diagnostic yield between adults aged <50 and ≥50 years in Oman using a large, bicenter cohort. Colonoscopy quality metrics were assessed to validate that observed age-group differences reflect true neoplasia burden rather than procedural variations. By quantifying age-specific rates of adenomas, advanced premalignant lesions (APLs), and cancers under standardized quality conditions, this work provides reliable local evidence to inform age-tailored CRC screening strategies and policy planning in the region.

## 2. Methods

### 2.1. Study Design and Setting

This cross-sectional study was conducted at two tertiary referral centers in Muscat, Oman: Sultan Qaboos University Hospital (SQUH) and the Royal Hospital (RH). Together, these institutions serve as national referral centers and provide comprehensive gastroenterology and endoscopy services to Oman’s population of approximately 4.6 million (2020 census) [10]. Both hospitals serve as the primary tertiary care facilities for all governorates of Oman, with catchment areas that extend nationwide. SQUH serves as the country’s main academic medical center and receives referrals from all regions, while RH is the largest governmental tertiary hospital and serves as the national referral center for complex medical and surgical cases. Collectively, these two institutions account for an estimated 60–70% of all tertiary-level colonoscopy procedures performed in Oman. All adult colonoscopy procedures performed between 1 January 2018 and 31 December 2021 were eligible for review. Ethical approval was obtained from the Medical Research Ethics Committee at the College of Medicine and Health Sciences, Sultan Qaboos University, prior to data collection (MREC code #2692).

### 2.2. Eligibility Criteria

All colonoscopies performed in adults aged ≥18 years during the study period were screened for inclusion, regardless of indication (symptomatic presentation or screening). Exclusion criteria included: (1) a documented diagnosis of inflammatory bowel disease (ulcerative colitis or Crohn’s disease), and (2) a prior diagnosis of CRC before the index colonoscopy. Patients with familial adenomatous polyposis (FAP), Lynch syndrome, or other known hereditary cancer syndromes were not excluded and were included in the analysis if they met the inclusion criteria. These criteria were applied to restrict the analysis to newly presenting cases and to avoid confounding from surveillance colonoscopies in patients with established IBD or known cancer.

### 2.3. Sampling Strategy and Sample Size

A stratified random sampling strategy was employed to ensure proportional representation by institution and calendar year. During the study period, 5638 colonoscopies were performed at RH and 2486 at SQUH. Based on prior studies reporting ADR of 18–25% in Middle Eastern populations and symptomatic cohorts [11,12], we assumed an adenoma prevalence of 20% for sample size estimation. With a 95% confidence level, a 5% margin of error, and a finite population correction, the minimum required sample sizes were estimated at 1190 procedures from RH and 950 from SQUH. A total of 2042 colonoscopy records were reviewed, with sampling proportional to institutional volume and year of procedure.

### 2.4. Data Collection and Variable Definitions

Data were extracted from electronic medical records and endoscopy reporting systems using a standardized data abstraction form. Collected variables included patient age, sex, region of residence, family history of CRC (first- or second-degree relative), and indication for colonoscopy, categorized as symptomatic, screening, surveillance, or incidental imaging findings. Colonoscopy indication was categorized as: (1) symptomatic—procedures performed for alarm symptoms (rectal bleeding, abdominal pain, change in bowel habits, weight loss, anemia) or concerning clinical findings; (2) screening—asymptomatic average-risk individuals undergoing opportunistic screening (as no organized population-based screening program exists in Oman, these represent individual physician-initiated or patient-requested screening rather than systematic program-based screening); (3) surveillance—follow-up colonoscopy after previous adenoma removal (post-polypectomy surveillance) or for individuals with high-risk family history requiring periodic endoscopic monitoring (post-cancer surveillance was excluded as per exclusion criteria); or (4) incidental findings on abdominal imaging requiring endoscopic evaluation.

Histopathological classification followed the 2019 World Health Organization criteria [13] and the U.S. Multi-Society Task Force guidelines [14]. Lesions were categorized as non-neoplastic polyps; conventional adenomas (tubular, tubulovillous, or villous); APL, defined as adenomas ≥10 mm, those with villous features or high-grade dysplasia, sessile serrated lesions ≥10 mm or with dysplasia, or traditional serrated adenomas; and CRC, defined as biopsy-proven invasive carcinoma. Advanced colorectal neoplasia (ACRN) was defined as the presence of APL and/or CRC.

### 2.5. Colonoscopy Quality Metrics

All procedures were performed by consultant gastroenterologists using high definition colonoscopes under moderate sedation or monitored anesthesia according to institutional protocols. Cecal intubation rate (CIR) was defined as successful intubation of the cecum, confirmed by visualization of the appendiceal orifice and ileocecal valve, and expressed as the proportion of completed examinations [8]. For incomplete procedures, the primary reason was documented.

ADR was defined as the proportion of colonoscopies in which at least one conventional adenoma was identified histologically [8,9]. ADR was calculated separately for patients aged <50 years and ≥50 years. Consistent with standard colonoscopy quality reporting practices, all procedures (both complete and incomplete colonoscopies) were included in the denominator for ADR and all neoplasia calculations (APL, CRC, ACRN). This intention-to-treat approach reflects real-world detection rates and aligns with guidelines from major gastroenterology societies. Bowel preparation quality was assessed by the endoscopist and categorized as adequate or inadequate based on mucosal visualization, consistent with institutional standards aligned with the Boston Bowel Preparation Scale framework [15]. CIR, ADR, and bowel preparation adequacy were compared across age groups. Quality metrics were assessed to ensure that age-group comparisons of adenoma detection were not confounded by differences in procedural completeness or preparation quality between younger and older patients. Given that ADR is our primary outcome, it was essential to validate that both age groups received comparable examination quality. Differences in cecal intubation rates or bowel preparation adequacy could artificially inflate or deflate ADR comparisons, leading to spurious conclusions about age-related neoplasia risk. These metrics, therefore, serve as validation measures to confirm that observed differences in neoplasia detection reflect true biological and risk differences rather than procedural artifacts.

### 2.6. Age Group Definition and Outcomes

Participants were categorized into age groups <50 years and ≥50 years to reflect internationally accepted CRC screening thresholds and facilitate comparison between early-onset and average-risk populations. The primary outcome was ADR. Secondary outcomes included the prevalence of APL, CRC, and ACRN; anatomical distribution of neoplasia (right colon, left colon, rectum); associations between presenting symptoms and neoplasia; and diagnostic yield, expressed as the number needed to scope (NNS), calculated as the inverse of APL or ACRN prevalence within each subgroup.

### 2.7. Statistical Analysis

Statistical analyses were performed using R software (version 4.2.3; R Foundation for Statistical Computing, Vienna, Austria). Continuous variables were summarized as medians with interquartile ranges and compared using the Mann–Whitney U test. Categorical variables were reported as frequencies and percentages and compared using Chi-square or Fisher’s exact tests as appropriate.

Associations between presenting symptoms and ACRN were evaluated using univariable logistic regression, stratified by age group, with results reported as odds ratios and 95% confidence intervals. This approach was selected to provide clinically interpretable estimates for symptom-based risk stratification without the added complexity of multivariable adjustment. Given the descriptive nature of the study and its focus on diagnostic yield rather than causal inference, priority was placed on transparent reporting of individual symptom associations that can be directly applied in clinical triage and endoscopic decision-making.

Statistical significance was defined as a two-tailed *p*-value < 0.05. Analyses were conducted using complete-case data, and sensitivity analyses were performed to assess the robustness of key findings.

## 3. Results

### 3.1. Baseline Characteristics

A total of 2041 colonoscopies were included, comprising 742 (36.3%) procedures in individuals aged <50 years and 1299 (63.7%) in those aged ≥50 years. The median age of the overall cohort was 56 years (IQR 43–67), with 38 years (IQR 30–44) in the younger group and 64 years (IQR 57–71) in the older group (*p* < 0.001). The sex distribution was comparable between age groups (male: 53.1% vs. 54.7%; *p* = 0.497). A family history of CRC was reported more frequently among younger patients (7.0%) than older patients (3.2%; *p* < 0.001), predominantly involving first-degree relatives.

Participants originated from all major governorates of Oman, with 45.0% residing in Muscat, where both participating tertiary hospitals are located. Given these institutions’ roles as national referral centers, particularly for complex or advanced gastrointestinal cases, this regional distribution reflects broad national representation rather than local clustering. Table 1 summarizes the baseline demographic and clinical characteristics of the study population.

### 3.2. Clinical Indications and Presenting Symptoms

The majority of colonoscopies were performed for symptomatic indications (58.8% overall), which were significantly more common among patients aged <50 years (65.1%) than among those aged ≥50 years (55.3%; *p* < 0.001).

Among younger adults, the most frequent presenting symptoms were bloody diarrhea (34.4%), abdominal pain (27.2%), and rectal bleeding (23.5%), whereas in the ≥50-year group, the predominant indications were anemia (19.1%) and constipation (16.9%).

Screening and surveillance colonoscopies accounted for 15.8% and 12.3% of all procedures, respectively, and were more frequently observed in older adults (19.2% screening and 14.6% surveillance in ≥50 years vs. 9.3% screening and 8.1% surveillance in <50 years, *p* < 0.001 for both). The majority of procedures (71.9%, n = 1467) were performed for symptomatic indications. Among screening colonoscopies, all represented opportunistic screening (individual physician-initiated or patient-requested) rather than organized program-based screening, as Oman currently lacks a population-based CRC screening program. Surveillance procedures were primarily for post-polypectomy follow-up (adenoma surveillance) or high-risk family history monitoring, with post-cancer surveillance excluded per study criteria. Despite 4.6% of all patients reporting a family history of CRC (Table 1), only 0.05% underwent colonoscopy with family history documented as the primary indication. This discrepancy reflects a substantial gap between recognition of hereditary risk and the use of colonoscopy for risk-based screening.

Patients were referred to both hospitals from across Oman; however, due to the tertiary referral nature of Sultan Qaboos University Hospital and Royal Hospital, the study cohort was predominantly symptomatic (71.9%, n = 1467), with only 15.8% undergoing screening and 12.3% undergoing surveillance procedures. This population composition likely results in higher neoplasia prevalence than would be observed in average-risk asymptomatic screening populations, as tertiary referral centers tend to receive patients with more severe symptoms, positive family history, and complex clinical presentations. Readers should interpret the absolute neoplasia rates in this context, recognizing that our findings are most directly applicable to symptomatic diagnostic colonoscopy rather than population-based screening settings. Table 2 presents the detailed distribution of colonoscopy indications and presenting symptoms by age group.

### 3.3. Colonoscopy Performance Metrics

Colonoscopy quality indicators are summarized in Table 3. High cecal intubation rates and adequate bowel preparation quality in both age groups confirm that the observed differences in ADR between younger and older adults reflect true differences in neoplasia prevalence rather than procedural quality variations. The overall CIR was 72.1%, with comparable rates between patients aged <50 years (74.3%) and those ≥50 years (70.8%; *p* = 0.096). These high cecal intubation rates demonstrate that our findings are not biased by differences in examination completeness across age groups. Similarly, bowel preparation adequacy was comparable across age groups (data shown in Table 3), ensuring that mucosal visualization quality did not confound neoplasia detection. ADR was 12.5% overall and significantly higher in those ≥50 years (14.9% vs. 8.5%, *p* < 0.001), validating that the age difference in adenoma detection reflects genuine biological risk differences rather than procedural factors.

Among the 570 incomplete procedures (27.9%), the most frequently documented reasons were inadequate bowel preparation (38.9%), undocumented causes (39.6%), and technical factors such as obstruction (7.5%) or looping/tortuosity (4.6%). Undocumented reasons were significantly more frequent among patients aged <50 years (48.2% vs. 35.4%; *p* = 0.003), whereas obstruction-related incompletion was more common in the ≥50 group (9.2% vs. 4.2%; *p* = 0.031). The high proportion of undocumented reasons for incomplete procedures (39.6%), particularly among younger patients (48.2%), represents a documentation quality gap that limits our ability to fully characterize procedural challenges and should be addressed through standardized endoscopy reporting protocols at both institutions.

A visual representation of the incomplete procedure by age group is provided in Supplementary Figure S1.

#### 3.3.1. Sensitivity Analysis: Symptomatic Colonoscopies Only

To ensure that the observed age-related differences were not driven by the inclusion of screening and surveillance colonoscopies, we performed a sensitivity analysis restricted to symptomatic procedures only (N = 1467, 71.9% of the total cohort). Among symptomatic patients, ADR remained significantly higher in those aged ≥50 years (16.2%) than in those aged <50 years (9.1%; *p* < 0.001). Similarly, ACRN rates were significantly higher in older symptomatic adults (14.8% vs. 6.4%, *p* < 0.001). The symptom-ACRN associations identified in the primary analysis—specifically rectal bleeding (OR = 2.17, *p* = 0.026), abdominal pain (OR = 2.14, *p* = 0.022), and loss of appetite (OR = 4.57, *p* = 0.045)—remained statistically significant in younger symptomatic adults when screening and surveillance cases were excluded. These findings confirm that the observed age-related disparities in neoplasia detection are not artifacts of including non-symptomatic indications but reflect genuine differences in disease burden across age groups.

#### 3.3.2. Histopathological Findings

Histopathological evaluation revealed age-dependent differences in colorectal lesion prevalence and type (Table 4). Conventional adenomas were significantly more frequent in patients aged ≥50 years than in those aged <50 years (14.9% vs. 8.5%; *p* < 0.001). Tubular adenomas constituted the majority (11.8% vs. 7.1%; *p* = 0.001), while tubulovillous/villous adenomas were less common but still more prevalent in older adults (3.1% vs. 1.3%; *p* = 0.022).

APL, including adenomas ≥10 mm, villous or high-grade dysplasia, and serrated lesions ≥10 mm or with dysplasia, were identified in 6.0% of all patients, with a higher rate in those ≥50 years (7.5% vs. 3.2%; *p* < 0.001). CRC was confirmed in 4.7% of all procedures, more common among older adults (5.8% vs. 2.7%; *p* = 0.002). When APL and CRC were combined, the prevalence of ACRN was 13.3% in the ≥50 group, compared with 5.9% in younger adults (*p* < 0.001).

**Figure 1:**
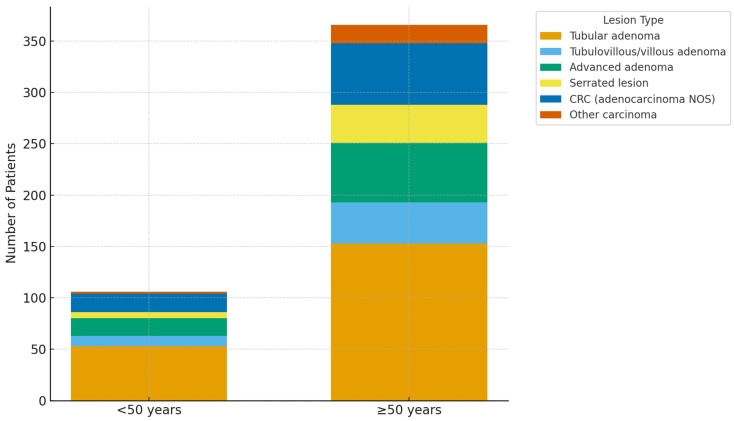
Age-related distribution of colorectal neoplastic lesions (<50 vs. ≥50 years). Stacked bar chart showing proportions of tubular, tubulovillous/villous, advanced adenomas, serrated lesions, and colorectal carcinoma (CRC) among patients in each age group. Lesion categories are not mutually exclusive; advanced adenomas are subsets of conventional adenomas. The higher prevalence of adenomas and carcinomas in the ≥50 group reflects the observed age-related neoplasia gradient (*p* < 0.001).

Anatomically, ACRN was distributed across the right colon (23.5%), left colon (22.1%), and rectum (22.1%) without significant variation by age group (*p* > 0.05). A visual summary of neoplastic distribution is presented in Figure 1.

### 3.4. Association Between Symptoms and Neoplasia

Univariable logistic regression demonstrated distinct symptom–neoplasia associations across age groups (Table 5). Among patients aged <50 years, *abdominal pain* (OR = 2.14; 95% CI: 1.15–3.98; *p* = 0.022), *rectal bleeding* (OR = 2.17; 95% CI: 1.15–4.08; *p* = 0.026), and *loss of appetite* (OR = 4.57; 95% CI: 1.23–17.02; *p* = 0.045) were significantly associated with ACRN.

In individuals aged ≥50 years, significant associations were observed for *rectal bleeding* (OR = 1.57; 95% CI: 1.07–2.31; *p* = 0.029), *nausea* (OR = 2.93; 95% CI: 1.19–7.22; *p* = 0.025), *loss of appetite* (OR = 3.86; 95% CI: 2.01–7.42; *p* < 0.001), and *weight loss* (OR = 1.94; 95% CI: 1.10–3.40; *p* = 0.025). Other symptoms—including chronic constipation, diarrhea, and anemia—were not significantly correlated with ACRN in either group (*p* > 0.05).

**Figure 2:**
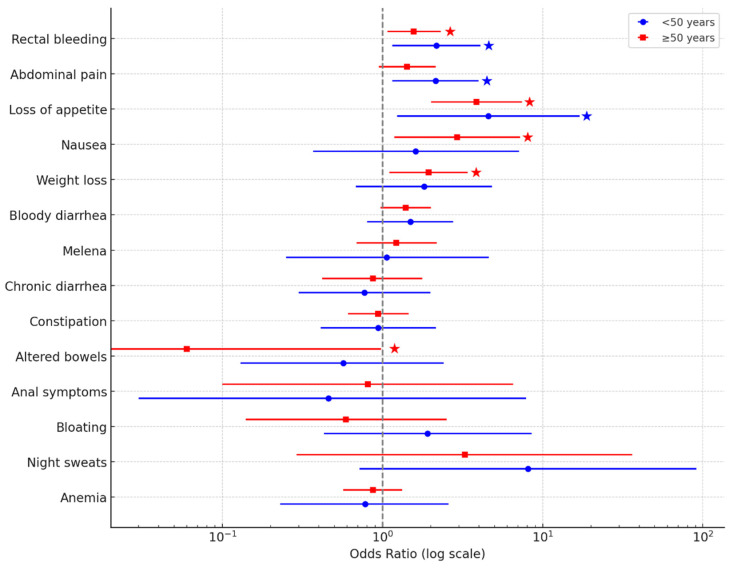
Forest plot of univariable associations between presenting symptoms and advanced colorectal neoplasia (ACRN), stratified by age group. Odds ratios (OR) with 95% confidence intervals (CI) are shown on a logarithmic scale for patients aged <50 years (blue circles) and ≥50 years (red squares). Stars (★) to the right of the CI bars denote statistically significant associations (*p* < 0.05). Among younger adults, abdominal pain, rectal bleeding, and loss of appetite showed the strongest associations with ACRN, while in older adults, rectal bleeding, nausea, loss of appetite, and weight loss were most predictive.

A forest plot illustrating these associations with corresponding odds ratios and confidence intervals is shown in Figure 2.

### 3.5. Diagnostic Yield and Efficiency

Subgroup analysis demonstrated that the diagnostic yield for APL and CRC varied by symptom and age group (Table S1). Among younger adults (<50 years), *rectal bleeding* had the highest CRC detection rate (4.6%), followed by *abdominal pain* (4.0%) and *bloody diarrhea* (3.5%). In older adults (≥50 years), yields were higher overall, with *rectal bleeding* (12.2%), *abdominal pain* (11.6%), and *bloody diarrhea* (10.2%) showing the greatest CRC detection rates (*p* < 0.05 for all vs. <50 years). For APL, age-related differences were smaller, but still favored the ≥50 group across most symptom categories.

A visual comparison of APL and CRC yields across symptoms and age groups is presented in Figure S2, which illustrates the proportional gradient in diagnostic efficiency.

### 3.6. Number Needed to Scope by Symptom

The diagnostic efficiency, expressed as number needed to scope (NNS) to detect one ACRN, varied substantially by symptom and age group (Table 6). Across all symptoms, NNS values were consistently lower among patients aged ≥50 years, confirming higher diagnostic efficiency with increasing age. For example, *loss of appetite* yielded the lowest NNS in both cohorts (4.7 in <50 years; 2.8 in ≥50 years), followed by *rectal bleeding* (10.2 vs. 5.5, respectively). In younger adults, *abdominal pain* (NNS = 12.1) and *bloody diarrhea* (NNS = 14.3) also demonstrated moderate efficiency, whereas *constipation* and *anemia* required substantially more procedures to detect a single ACRN (NNS > 25). In contrast, older adults showed the greatest efficiency for *loss of appetite*, *rectal bleeding*, and *weight loss* (all NNS < 6). A visual summary of these differences is presented in Figure S3.

## 4. Discussion

This large bicenter cross-sectional study provides one of the most comprehensive evaluations to date of age-related differences in colorectal neoplasia among adults in Oman. Using data from more than 2000 colonoscopies performed at two national referral centers, we demonstrate a clear age-dependent gradient in neoplastic findings, with substantially higher rates of adenomas, APL, CRC, and ACRN among individuals aged ≥50 years compared with those <50 years. Importantly, however, clinically significant neoplasia was also identified in a meaningful proportion of younger adults, supporting growing concerns about EOCRC and underscoring the need for age- and risk-adapted diagnostic strategies.

Beyond age, symptom profile emerged as a critical determinant of diagnostic yield among symptomatic patients. In younger adults presenting with symptoms, rectal bleeding (OR 2.17, 95% CI 1.15–4.08, *p* = 0.026) and abdominal pain (OR 2.14, 95% CI 1.15–3.98, *p* = 0.022) showed significant associations with ACRN. These symptom-ACRN associations remained significant when we restricted the analysis to symptomatic colonoscopies only (excluding screening and surveillance cases), confirming their validity for clinical triage of symptomatic younger individuals. These findings have direct implications for prioritizing colonoscopy access among younger adults with alarm symptoms and are distinct from considerations for asymptomatic screening populations. While screening and surveillance colonoscopies comprised 28.1% of our cohort, sensitivity analyses demonstrated that age-related differences in neoplasia detection persist across all indication categories (symptomatic, screening, and surveillance), reflecting genuine biological risk differences rather than differential indication patterns.

### 4.1. EOCRC in Global and Regional Perspective

The burden of EOCRC observed in this cohort reflects a broader global epidemiologic shift. Worldwide, CRC incidence among individuals younger than 50 years has nearly doubled since the 1990s [16], contrasting with declining rates in older adults, largely attributed to effective population-based screening programs. In the United States, CRC incidence in adults aged 20–49 continues to increase by approximately 1.5% annually [17], with projections suggesting that by 2030, nearly one-quarter of rectal cancers will occur in this age group [18].

In the Middle East, and particularly in Oman, this shift appears even more pronounced [19]. CRC is now the most common cancer among Omani men and the second most common among women, with a steadily rising incidence over the past two decades [4]. Notably, approximately 20% of cases occur before age 40, and nearly one-third are diagnosed before age 50 in some institutional series [4], a proportion substantially higher than that reported in most Western populations. This apparent early-age burden is likely amplified by the absence of organized screening among older adults, resulting in delayed diagnoses across all age groups and a relative overrepresentation of EOCRC.

National data show that nearly half of CRC cases are diagnosed at stage IV [4], further underscoring the consequences of symptom-based detection. Within this context, our age-group comparison of colonoscopy findings provides essential local evidence to inform early detection strategies and highlight EOCRC as a growing public health challenge in Oman and the wider region.

### 4.2. Validation of Procedural Quality Across Age Groups

Before interpreting age-related differences in adenoma detection, we must ensure procedural quality was comparable across age groups. Our quality metrics demonstrate that examination completeness and preparation adequacy did not differ substantially across age strata. Cecal intubation rates exceeded 70% in both younger (<50 years: 74.3%) and older (≥50 years: 70.8%) adults (*p* = 0.096), confirming successful visualization of the full colonic mucosa across age groups. Similarly, bowel preparation quality was adequate and comparable between groups (Table 3), ensuring sufficient mucosal visualization for polyp detection in both populations. Some studies have suggested that younger patients may achieve superior bowel preparation due to differences in dietary patterns or compliance behaviors; however, we observed no such difference in our cohort. These comparable quality metrics validate that the lower ADR in younger adults (8.5% vs. 14.9% in older adults, *p* < 0.001) genuinely reflects lower baseline adenoma prevalence rather than suboptimal examination quality or incomplete visualization. This confirmation is critical for the reliability of our age-stratified findings and strengthens confidence in using these data to inform screening policy decisions, as the observed differences represent true biological risk variation rather than procedural artifacts.

### 4.3. Colonoscopy Quality and Adenoma Detection Rate

ADR serves as a validated surrogate marker of colonoscopy quality and a key determinant of CRC prevention. As expected, ADR was significantly higher among individuals aged ≥50 years, reflecting the well-established increase in adenoma prevalence with age. Despite the predominantly symptomatic nature of this cohort, the ADR observed in older adults likely meets or exceeds internationally accepted quality benchmarks for average-risk populations [8], suggesting satisfactory endoscopic performance across participating centers.

These findings align with international screening data. Large population-based studies have demonstrated substantially higher adenoma and advanced neoplasia detection rates in individuals aged ≥50 compared with younger adults [20,21], with corresponding improvements in diagnostic efficiency and lower numbers needed to scope (NNS). The favorable ADR observed in this study provides indirect reassurance regarding procedural quality, even in the absence of comprehensive reporting of additional quality metrics such as withdrawal time.

Conversely, the lower ADR in younger adults reflects their lower baseline risk and underscores the importance of careful patient selection in this group. Routine colonoscopy for low-risk younger individuals is unlikely to be cost-effective; however, as demonstrated in this study, targeted colonoscopy based on symptom stratification can yield clinically meaningful findings.

### 4.4. Symptom Profiles and Diagnostic Yield in Younger Adults

In Oman, where CRC screening is not routinely implemented, colonoscopy remains largely symptom-driven. Our findings show that younger adults are more likely to present with alarm symptoms, and that specific symptoms, particularly rectal bleeding and abdominal pain, are strongly associated with ACRN in this age group. These results are consistent with international evidence identifying hematochezia and abdominal pain as common and predictive features of EOCRC [22].

Importantly, diagnostic yield increased further when lower gastrointestinal symptoms were accompanied by constitutional features such as unexplained weight loss or loss of appetite. This aligns with existing guidance emphasizing the significance of systemic symptoms and iron-deficiency anemia in younger patients [22]. In contrast, colonoscopy performed for vague or non-alarm symptoms demonstrated a low yield for neoplasia, supporting a more selective diagnostic approach in truly low-risk presentations [23].

Historically, limited endoscopic evaluation—such as flexible sigmoidoscopy—was considered adequate for younger patients with mild rectal bleeding [24,25]. However, the rising incidence of EOCRC and the increasing recognition of proximal and advanced lesions in younger adults challenge this paradigm. Our findings support a proactive diagnostic strategy: young adults with persistent rectal bleeding, abdominal pain, unexplained weight loss, or anemia should undergo full colonoscopic evaluation, as the probability of advanced neoplasia is sufficiently high to justify comprehensive assessment.

### 4.5. Implications for Early Detection and Screening Policy in Oman

Based on our findings in this symptomatic cohort, we can confidently make several recommendations for current clinical practice in Oman. First, clinicians should maintain a low threshold for diagnostic colonoscopy in symptomatic adults under 50 years presenting with alarm features—particularly rectal bleeding, abdominal pain, or constitutional symptoms—as these symptoms showed significant associations with ACRN (5.9% ACRN rate in the <50 age group). Second, risk-stratified diagnostic approaches prioritizing individuals with positive family history, persistent or severe symptoms, or multiple alarm features are supported by our data and can be implemented immediately without requiring an organized screening infrastructure. Third, the substantial burden of advanced neoplasia in younger symptomatic adults (2.7% CRC rate in <50 years) reinforces that EOCRC should be considered in the differential diagnosis and not dismissed based on age alone. These diagnostic recommendations are directly supported by our data and are actionable in current clinical practice, independent of future screening program decisions.

Regarding the development of screening policy, the pronounced age-related differences in neoplasia prevalence observed in this study have important implications for Oman. At present, the lack of a national screening program leads to late-stage, symptom-based diagnosis for most patients. The high prevalence of ACRN and favorable diagnostic efficiency among adults aged ≥50 years strongly support prioritizing this group for organized screening.

Globally, screening recommendations have shifted in response to EOCRC trends. The American Cancer Society now recommends initiating average-risk screening at age 45, and Middle East consensus guidelines have adopted similar thresholds [26]. Some regional programs, including in the United Arab Emirates, recommend screening from age 40 using fecal immunochemical testing (FIT) or colonoscopy [27].

While our findings from this predominantly symptomatic cohort cannot directly determine optimal screening strategies, they provide evidence that could inform future screening program development. Should Oman establish organized screening, a stepwise, resource-conscious approach merits consideration, potentially involving FIT-based screening for average-risk adults with colonoscopy reserved for positive tests. Such an approach would align with international best practices and be feasible within existing healthcare infrastructure [4]. The specific screening age threshold (45, 50, or another age) would need to be determined through prospective screening trials and cost-effectiveness modeling tailored to Oman’s healthcare context and population. Simultaneously, maintaining a low threshold for diagnostic colonoscopy in symptomatic individuals under 50, particularly those with alarm features, could help mitigate delayed diagnoses and reduce the proportion presenting at advanced stages.

Implementing national CRC screening must be balanced against available healthcare resources. Oman’s health system would require expanding endoscopic capacity, strengthening laboratory support, and launching public awareness initiatives to achieve meaningful screening uptake—challenges that have similarly constrained regional programs [2,28]. Risk-stratified approaches may further enhance efficiency by prioritizing individuals with a family history of CRC or younger men, given evidence of more rapid increases in EOCRC incidence in males [21]. More broadly, these findings reinforce the need for Middle Eastern countries to recalibrate CRC screening strategies to reflect regional demographics and disease patterns rather than relying solely on Western benchmarks.

An important caveat when interpreting these findings for screening policy is that our cohort was predominantly symptomatic (71.9%) and derived from tertiary referral centers, which likely results in higher neoplasia prevalence than would be expected in an asymptomatic general population screening setting. In organized screening programs enrolling average-risk asymptomatic individuals, ADR and advanced neoplasia prevalence would be expected to be lower than the rates observed in our symptomatic cohort. Therefore, while our age-stratified findings demonstrate substantial disease burden in younger symptomatic adults and support the need for age-tailored strategies, the absolute neoplasia rates reported here should not be directly extrapolated to asymptomatic screening populations. Instead, our data provide evidence supporting: (1) the importance of prompt colonoscopic evaluation for symptomatic younger adults, particularly those with alarm symptoms; (2) the age-related gradient in neoplasia risk that would likely persist (though at lower absolute rates) in screening populations; and (3) the need for prospective screening trials in Oman to determine true detection rates in asymptomatic average-risk populations. The tertiary referral nature of our centers means our cohort may be enriched with complex cases, family history-positive individuals, and patients with more severe or persistent symptoms compared to primary care populations. This selection bias strengthens the clinical applicability of our symptom-based risk stratification findings but limits direct application to screening program planning without adjustment for population differences.

An important consideration for Oman’s screening policy is determining the optimal age threshold for initiating organized screening. While our study used age 50 as the primary cutoff to align with traditional international thresholds and ensure adequate statistical power, this may not represent the optimal threshold for Oman’s specific context. Given the younger population structure in Oman and the Middle East compared to Western countries, and the documented high burden of EOCRC (30–35% of cases occurring before age 50), there is a strong rationale for considering earlier screening initiation, potentially at age 45 or even 40 years. Recent updates to international guidelines, including the American Cancer Society (2018) [26] and U.S. Preventive Services Task Force (2021) [14] recommendations to begin average-risk screening at age 45, support this trend toward earlier screening. However, our current sample size precluded robust statistical analysis using finer age stratification (e.g., comparing <40, 40–44, 45–49, and ≥50 age groups), as subdividing the younger cohort would yield subgroups with insufficient numbers for reliable symptom-specific and multivariable analyses. Determining the optimal screening age threshold for Oman will require larger-scale epidemiological studies with adequate power for granular age stratification, combined with health economic modeling to assess cost-effectiveness at different age cutoffs and feasibility analyses considering healthcare infrastructure capacity. Our findings provide foundational evidence that a substantial neoplasia burden exists in adults under 50 years, supporting the need for age-tailored screening strategies, but the precise age threshold (whether 40, 45, or 50 years) should be determined through dedicated prospective screening program pilot studies and implementation research specific to Oman’s healthcare context.

### 4.6. Strengths and Limitations

The strengths of this study include its large sample size, bicenter design, and inclusion of two national referral hospitals, enhancing generalizability across Oman. The direct comparison of age groups and integration of symptom profiles with histopathological outcomes provide clinically actionable insights for endoscopic decision-making.

Several important limitations must be considered. First, our study population was predominantly symptomatic (71.9%) and derived from two tertiary referral centers, which has significant implications for generalizability to screening populations. This referral bias likely resulted in higher neoplasia prevalence than would be observed in average-risk asymptomatic individuals undergoing organized screening. Consequently, the absolute ADR and ACRN rates reported here (12.5% overall ADR; 5.9% ACRN in <50 years, 13.3% in ≥50 years) should not be directly applied to screening program planning without recognizing that asymptomatic screening populations would likely demonstrate lower detection rates. This limitation particularly affects the interpretation of cost-effectiveness and number-needed-to-screen estimates, which would differ substantially between symptomatic tertiary care populations and asymptomatic screening cohorts. Our findings are most directly applicable to: (a) informing diagnostic strategies for symptomatic patients at tertiary centers, (b) understanding relative age-related differences that would likely persist in screening settings, and (c) justifying the need for prospective screening program trials in Oman to establish true population-based detection rates.

The high rate of undocumented reasons for incomplete colonoscopies (39.6%) represents a quality documentation gap that limited our ability to comprehensively analyze procedural barriers. This finding highlights the need for mandatory structured reporting fields in endoscopy documentation systems to ensure complete capture of incomplete procedure etiologies—an actionable institutional improvement that would enhance quality monitoring and targeted interventions for specific procedural challenges.

Additional limitations include the retrospective design and reliance on existing documentation, which may introduce reporting bias and limit the assessment of certain quality indicators. Beyond the symptomatic nature of the cohort, referral bias is possible given the tertiary-care setting, as these national referral centers tend to receive patients with more severe symptoms, a positive family history, and complex clinical presentations than primary care populations.

Molecular or genetic data were not available to characterize EOCRC biology. In addition, while the sample size was substantial for the primary age comparison (<50 vs. ≥50 years), it was insufficient to support robust analyses using finer age stratification at alternative cutoffs such as 40 or 45 years. Symptom-ACRN associations were assessed using univariable logistic regression without multivariable adjustment for potential confounders such as sex, family history, or age within strata. While this approach provides clinically interpretable associations for symptom-based triage, it does not account for potential confounding. The reported odds ratios should therefore be interpreted as descriptive measures of association rather than independent causal effects. Future studies with larger sample sizes could employ multivariable modeling to assess the independent effects of symptoms while adjusting for demographic and clinical factors. Future larger-scale multicenter studies with adequate statistical power for granular age-specific analyses would help refine optimal screening age thresholds for Oman and the wider Middle East region. Nonetheless, the consistency of these findings with international data supports their validity and relevance.

## 5. Conclusions

This large bicenter study demonstrates clear age-related disparities in colorectal neoplasia among Omani adults, with significantly higher rates of adenomas, APLs, and CRC in individuals aged ≥50 years compared to younger adults. Despite lower overall neoplasia rates in the younger age group, this study identified a substantial and clinically important burden of ACRN in symptomatic individuals under 50 years. Among younger adults, rectal bleeding, abdominal pain, and constitutional symptoms were significantly associated with ACRN and can help identify high-risk individuals who warrant colonoscopic evaluation. While these symptoms are important indicators across all age groups, their identification in younger patients has particular clinical utility for risk stratification in settings with limited organized screening programs.

These findings provide local evidence on age-specific neoplasia burden and symptom-based risk stratification that can inform the development of age-tailored CRC screening and early detection strategies in Oman and similar Middle Eastern settings. However, as our cohort was predominantly symptomatic (71.9%) and derived from tertiary referral centers, detection rates in asymptomatic screening populations may differ from those reported here. Therefore, while our data support the need for organized screening programs and provide evidence for age-stratified approaches, prospective screening trials are essential to establish true population-based detection rates in asymptomatic average-risk individuals. Future studies should incorporate prospective data collection with standardized documentation protocols to ensure complete capture of procedural quality indicators, including reasons for incomplete examinations and comprehensive quality metrics. Such prospective research evaluating screening program implementation, incorporating molecular profiling, and providing longitudinal follow-up data is needed to further refine region-specific prevention policies and determine optimal screening age thresholds for this population.

## Figures and Tables

**Table 1:** Baseline demographic and clinical characteristics of patients undergoing colonoscopy, stratified by age group (<50 vs. ≥50 years; N = 2041).

Characteristic	Total (N = 2041)	<50 Years (n = 742)	≥50 Years (n = 1299)	*p*-Value
Demographics				
Age, median (IQR)	56.0 (43.0–67.0)	38.0 (30.0–44.0)	64.0 (57.0–71.0)	**<0.001**
Sex, male	1104 (54.1%)	394 (53.1%)	710 (54.7%)	0.497
Sex, female	937 (45.9%)	348 (46.9%)	589 (45.3%)	
Family History of CRC				
Any family history	94 (4.6%)	52 (7.0%)	42 (3.2%)	**<0.001**
First-degree relative	70 (3.4%)	37 (5.0%)	33 (2.5%)	0.412
Second-degree relative	12 (0.6%)	7 (0.9%)	5 (0.4%)	0.822
First and second-degree	12 (0.6%)	8 (1.1%)	4 (0.3%)	0.397
Region of Residence				
Muscat	918 (45.0%)	315 (42.5%)	603 (46.4%)	0.083
Al Batinah	512 (25.1%)	195 (26.3%)	317 (24.4%)	0.347
Ad Dakhiliyah	216 (10.6%)	84 (11.3%)	132 (10.2%)	**0.009**
Ash Sharqiyah	177 (8.7%)	77 (10.4%)	100 (7.7%)	**0.039**
Dhofar	71 (3.5%)	22 (3.0%)	49 (3.8%)	0.338
Ad Dhahirah	106 (5.2%)	30 (4.0%)	76 (5.9%)	0.077
Other (combined <1%)	41 (2.0%)	19 (2.6%)	22 (1.7%)	0.148

Values are presented as median (interquartile range) for continuous variables and n (%) for categorical variables. Comparisons between age groups were performed using the Mann–Whitney U test for continuous variables and the chi-square test or Fisher’s exact test for categorical variables, as appropriate. Bolded *p*-values indicate statistical significance (*p* < 0.05). CRC = colorectal cancer; IQR = interquartile range.

**Table 2:** Clinical indications and presenting symptoms for colonoscopy stratified by age group (<50 vs. ≥50 years; N = 2041).

**A. Colonoscopy Indications**					
**Indications**		**Total (N = 2041)**	**<50 Years (n = 742)**	**≥50 Years (n = 1299)**	***p*-Value**
Symptomatic		1201 (58.8%)	483 (65.1%)	718 (55.3%)	**<0.001**
Surveillance/Follow-up		556 (27.2%)	153 (20.6%)	403 (31.0%)	**<0.001**
Abnormal imaging (CT)		112 (5.5%)	39 (5.3%)	73 (5.6%)	0.729
Screening		96 (4.7%)	36 (4.9%)	60 (4.6%)	0.811
Positive stool occult blood		13 (0.6%)	4 (0.5%)	9 (0.7%)	0.779
Family history (positive)		1 (0.05%)	0 (0.0%)	1 (0.1%)	1.000
Missing/Unspecified		62 (3.0%)	27 (3.6%)	35 (2.7%)	0.232
**B. Presenting Symptoms**					
**Symptom category**	**Symptom**	**Total (N = 2041)**	**<50 years (n = 742)**	**≥50 years (n = 1299)**	***p*** **-value**
GI Bleeding	Bloody diarrhea	540 (26.5%)	255 (34.4%)	285 (21.9%)	**<0.001**
	Rectal bleeding	395 (19.4%)	174 (23.5%)	221 (17.0%)	**<0.001**
	Melena	128 (6.3%)	32 (4.3%)	96 (7.4%)	**0.006**
Bowel Habit Changes	Chronic diarrhea	181 (8.9%)	105 (14.2%)	76 (5.9%)	**<0.001**
	Chronic constipation	344 (16.9%)	124 (16.7%)	220 (16.9%)	0.896
	Altered bowel movements	107 (5.2%)	56 (7.5%)	51 (3.9%)	**<0.001**
Abdominal Symptoms	Abdominal pain	401 (19.6%)	202 (27.2%)	199 (15.3%)	**<0.001**
	Abdominal distension/bloating	43 (2.1%)	19 (2.6%)	24 (1.8%)	0.281
	Nausea	45 (2.2%)	22 (3.0%)	23 (1.8%)	0.077
Constitutional Symptoms	Weight loss	128 (6.3%)	51 (6.9%)	77 (5.9%)	0.397
	Loss of appetite	56 (2.7%)	14 (1.9%)	42 (3.2%)	0.073
	Night sweats	6 (0.3%)	3 (0.4%)	3 (0.2%)	0.674
Other	Anal symptoms	25 (1.2%)	16 (2.2%)	9 (0.7%)	**0.004**
	Anemia	311 (15.2%)	63 (8.5%)	248 (19.1%)	**<0.001**

Values are presented as n (%). Statistical comparisons between age groups were performed using the chi-square test or Fisher’s exact test, as appropriate. Bolded *p*-values indicate statistical significance (*p* < 0.05).

**Table 3:** Colonoscopy quality indicators and completion outcomes stratified by age group (<50 vs. ≥50 years; N = 2041).

Quality Indicator	Total (N = 2041)	<50 Years (n = 742)	≥50 Years (n = 1299)	*p*-Value
Cecal Intubation Rate (CIR)	1471 (72.1%)	551 (74.3%)	920 (70.8%)	0.096
Adenoma Detection Rate (ADR)	256 (12.5%)	63 (8.5%)	193 (14.9%)	**<0.001**
Reasons for Incomplete Colonoscopy	n = 570 (27.9%)	n = 191 (25.7%)	n = 379 (29.2%)	
Not reported	226 (39.6%)	92 (48.2%)	134 (35.4%)	**0.003**
Inadequate bowel preparation	222 (38.9%)	67 (35.1%)	155 (40.9%)	0.179
Technical: obstruction/mass	43 (7.5%)	8 (4.2%)	35 (9.2%)	**0.031**
Technical: looping/tortuosity	26 (4.6%)	7 (3.7%)	19 (5.0%)	0.466
Discomfort or intolerance	23 (4.0%)	7 (3.7%)	16 (4.2%)	0.750
Other technical reasons	19 (3.3%)	8 (4.2%)	11 (2.9%)	0.419
Procedure limitation: sigmoidoscopy	7 (1.2%)	1 (0.5%)	6 (1.6%)	0.433
Anastomotic issues	4 (0.7%)	1 (0.5%)	3 (0.8%)	1.000

Values are presented as n (%). Cecal intubation rate (CIR) was defined as successful visualization of the cecum documented in the endoscopy report [8]. Adenoma detection rate (ADR) was defined as the proportion of colonoscopies detecting ≥1 conventional adenoma [8]. Comparisons between groups were performed using the chi-square test or Fisher’s exact test, as appropriate. Bolded *p*-values indicate statistical significance (*p* < 0.05).

**Table 4:** Histopathological spectrum of colorectal lesions stratified by age group (<50 vs. ≥50 years; N = 2041).

Histopathology	Total (N = 2041)	<50 Years (n = 742)	≥50 Years (n = 1299)	*p*-Value
Non-polyp benign findings	1545 (75.7%)	615 (82.9%)	930 (71.6%)	<0.001
Non-neoplastic polyps (e.g., Hyperplastic polyps, Inflammatory Polyps, Hamartomatous Polyps, Juvenile polyps, Lymphoid Polyps)	119 (5.8%)	47 (6.3%)	72 (5.5%)	0.525
Conventional adenomas	256 (12.5%)	63 (8.5%)	193 (14.9%)	<0.001
Tubular adenomas	206 (10.1%)	53 (7.1%)	153 (11.8%)	0.001
Tubulovillous/villous adenomas	50 (2.4%)	10 (1.3%)	40 (3.1%)	0.022
Advanced premalignant lesions (APL)	122 (6.0%)	24 (3.2%)	98 (7.5%)	<0.001
Advanced adenomas	75 (3.7%)	17 (2.3%)	58 (4.5%)	0.017
Serrated lesions	43 (2.1%)	6 (0.8%)	37 (2.8%)	0.003
Carcinomas				
All colorectal carcinomas	95 (4.7%)	20 (2.7%)	75 (5.8%)	0.002
Adenocarcinoma (NOS)	78 (3.8%)	18 (2.4%)	60 (4.6%)	0.018
Mucinous adenocarcinoma	2 (0.1%)	0 (0.0%)	2 (0.2%)	0.537
Signet ring cell carcinoma	1 (0.05%)	1 (0.1%)	0 (0.0%)	0.364
Undifferentiated carcinoma	6 (0.3%)	1 (0.1%)	5 (0.4%)	0.426
Intramucosal carcinoma	8 (0.4%)	0 (0.0%)	8 (0.6%)	0.057
Advanced colorectal neoplasia (ACRN) (APL + CRC)	217 (10.6%)	44 (5.9%)	173 (13.3%)	<0.001
**Tumor location among patients with ACRN (n = 217)**				
Right colon	51 (23.5%)	6 (13.6%)	45 (26.0%)	0.093
Left colon	48 (22.1%)	11 (25.0%)	37 (21.4%)	0.759
Rectum	48 (22.1%)	13 (29.5%)	35 (20.2%)	0.238
Unspecified/Unassigned	70 (32.3%)	14 (31.8%)	56 (32.4%)	1.000

Values are shown as n (%). Advanced premalignant lesions (APL) were defined per the WHO (2022) [13] and the U.S. Multi-Society Task Force (2021) [14] criteria as adenomas ≥ 10 mm, adenomas with villous features or high-grade dysplasia, and serrated lesions ≥ 10 mm or with dysplasia. Advanced colorectal neoplasia (ACRN) represents APL ± colorectal carcinoma. Statistical comparisons were performed using chi-square or Fisher’s exact tests. Abbreviations: APL = advanced premalignant lesion; ACRN = advanced colorectal neoplasia; CRC = colorectal cancer; NOS = not otherwise specified.

**Table 5:** Univariable association between presenting symptoms and advanced colorectal neoplasia (ACRN), stratified by age group (<50 vs. ≥50 years).

Symptom Category	Symptom	<50 Years OR (95% CI)	*p*-Value	≥50 Years OR (95% CI)	*p*-Value
GI Bleeding	Rectal bleeding	2.17 (1.15–4.08)	**0.026**	1.57 (1.07–2.31)	**0.029**
	Bloody diarrhea	1.49 (0.80–2.76)	0.251	1.39 (0.97–2.00)	0.076
	Melena	1.06 (0.25–4.59)	0.714	1.22 (0.69–2.18)	0.531
Bowel Habit Changes	Chronic diarrhea	0.77 (0.30–1.99)	0.823	0.87 (0.42–1.77)	0.862
	Chronic constipation	0.94 (0.41–2.16)	1.000	0.94 (0.61–1.45)	0.828
	Altered bowel movements	0.57 (0.13–2.41)	0.766	0.06 (0.00–0.98)	**0.001**
Abdominal Symptoms	Abdominal pain	2.14 (1.15–3.98)	**0.022**	1.42 (0.95–2.15)	0.090
	Abdominal distension/bloating	1.91 (0.43–8.53)	0.312	0.59 (0.14–2.52)	0.760
	Nausea	1.61 (0.37–7.14)	0.379	2.93 (1.19–7.22)	0.025
Constitutional	Weight loss	1.82 (0.68–4.83)	0.217	1.94 (1.10–3.40)	**0.025**
	Loss of appetite	4.57 (1.23–17.02)	**0.045**	3.86 (2.01–7.42)	**<0.001**
	Night sweats	8.09 (0.72–91.03)	0.168	3.27 (0.29–36.23)	0.349
Other	Anal symptoms	0.46 (0.03–7.87)	0.616	0.81 (0.10–6.54)	1.000
	Anemia	0.78 (0.23–2.59)	1.000	0.87 (0.57–1.33)	0.604

Odds ratios (OR) and 95% confidence intervals (CI) were derived from univariable logistic regression models within each age group. ACRN includes advanced premalignant lesions and colorectal carcinoma. Bolded *p*-values indicate statistical significance (*p* < 0.05). Symptoms were categorized thematically to reflect typical clinical presentations. Abbreviations: ACRN = advanced colorectal neoplasia; GI = gastrointestinal; OR = odds ratio; CI = confidence interval.

**Table 6:** Number needed to scope (NNS) to detect one advanced colorectal neoplasia (ACRN), stratified by presenting symptom and age group (<50 vs. ≥50 years).

Symptom Category	Symptom	<50 Years N	APL Prev % (95% CI)	NNS for APL (95% CI)	ACRN Prev % (95% CI)	NNS for ACRN (95% CI)	≥50 Years N	APL Prev % (95% CI)	NNS for APL (95% CI)	ACRN Prev % (95% CI)	NNS for ACRN (95% CI)
GI Bleeding	Rectal bleeding	174	5.2 (2.7–9.5)	19.3 (10.5–36.4)	9.8 (6.2–15.1)	10.2 (6.6–16.2)	221	5.9 (3.5–9.8)	17.0 (10.2–28.8)	18.1 (13.6–23.7)	5.5 (4.2–7.4)
	Bloody diarrhea	255	3.9 (2.1–7.1)	25.5 (14.2–46.6)	7.5 (4.8–11.3)	13.4 (8.8–20.7)	285	6.3 (4.0–9.8)	15.8 (10.2–24.8)	16.5 (12.6–21.2)	6.1 (4.7–7.9)
	Melena	32	6.2 (1.7–20.1)	16.0 (5.0–57.8)	6.2 (1.7–20.1)	16.0 (5.0–57.8)	96	10.4 (5.8–18.1)	9.6 (5.5–17.4)	15.6 (9.7–24.2)	6.4 (4.1–10.3)
Bowel Habit Changes	Chronic diarrhea	105	3.8 (1.5–9.4)	26.2 (10.7–67.1)	4.8 (2.1–10.7)	21.0 (9.4–48.8)	76	6.6 (2.8–14.5)	15.2 (6.9–35.2)	11.8 (6.4–21.0)	8.4 (4.8–15.7)
	Chronic constipation	124	2.4 (0.8–6.9)	41.3 (14.6–121.0)	5.6 (2.8–11.2)	17.7 (8.9–36.2)	220	4.5 (2.5–8.2)	22.0 (12.2–40.2)	12.7 (9.0–17.8)	7.9 (5.6–11.2)
	Altered bowel movements	56	0.0 (0.0–6.4)	NA	3.6 (1.0–12.1)	28.0 (8.3–101.5)	51	0.0 (0.0–7.0)	NA	0.0 (0.0–7.0)	NA
Abdominal Symptoms	Abdominal pain/discomfort	202	5.4 (3.1–9.5)	18.4 (10.5–32.6)	9.4 (6.1–14.2)	10.6 (7.0–16.4)	199	5.5 (3.1–9.6)	18.1 (10.4–32.1)	17.1 (12.5–22.9)	5.9 (4.4–8.0)
	Abdominal distension	19	0.0 (0.0–9.0)	NA	10.5 (2.9–32.3)	9.5 (3.1–34.1)	24	4.2 (0.7–20.3)	24.0 (4.9–137.6)	4.2 (0.7–20.3)	24.0 (4.9–137.6)
	Nausea	22	4.5 (0.8–21.8)	22.2 (4.6–121.4)	4.5 (0.8–21.8)	22.2 (4.6–121.4)	23	4.3 (0.8–21.2)	23.3 (4.7–128.5)	26.1 (11.8–48.5)	3.8 (2.1–8.5)
Constitutional Symptoms	Loss of appetite	14	7.1 (1.3–31.5)	14.0 (3.2–78.6)	21.4 (7.6–47.6)	4.7 (2.1–13.2)	42	14.3 (6.7–27.8)	7.0 (3.6–14.9)	35.7 (23.0–50.8)	2.8 (2.0–4.3)
	Weight loss	51	2.0 (0.3–11.0)	50.0 (9.1–333.3)	7.8 (3.1–18.5)	12.8 (5.4–32.3)	77	6.5 (3.2–12.7)	15.4 (7.9–31.3)	15.6 (9.7–24.2)	6.4 (4.1–10.3)
Other	Anemia	63	3.2 (0.9–10.9)	31.5 (9.2–114.3)	4.8 (1.6–13.1)	21.0 (7.6–61.3)	248	8.9 (5.9–13.1)	11.3 (7.7–16.9)	12.1 (8.6–16.7)	8.3 (6.0–11.6)
	Screening/Surveillance	189	2.6 (1.1–6.0)	37.8 (16.5–88.1)	4.8 (2.5–8.8)	21.0 (11.4–39.6)	463	8.2 (6.0–11.1)	12.2 (9.0–16.6)	12.3 (9.6–15.6)	8.1 (6.4–10.4)
	Overall	742	3.2 (2.2–4.8)	30.9 (21.0–45.8)	5.9 (4.4–7.9)	16.9 (12.7–22.5)	1299	7.5 (6.2–9.1)	13.3 (11.0–16.1)	13.3 (11.6–15.3)	7.5 (6.5–8.6)

Values represent the number of colonoscopies required to detect one ACRN (advanced premalignant lesion ± colorectal carcinoma) within each symptom subgroup. NNS was calculated as the inverse of ACRN prevalence. Lower values denote greater diagnostic efficiency. Abbreviations: ACRN = advanced colorectal neoplasia; NNS = number needed to scope.

## Data Availability

The data supporting the findings of this study are available from the corresponding author upon reasonable request.

## Supplementary Materials

The following supporting information can be downloaded at: https://www.mdpi.com/article/10.3390/medicina62020374/s1. Table S1. Symptom-specific diagnostic yield for advanced premalignant lesions (APL) and colorectal cancer (CRC), stratified by age group (<50 vs. ≥50 years). Figure S1. Distribution of reasons for incomplete colonoscopy by age group (<50 vs. ≥50 years). Bars represent the number of incomplete procedures attributed to each cause. Asterisks (*) denote statistically significant differences between age groups (*p* < 0.05). The two most common causes were undocumented reasons and inadequate bowel preparation, with significantly higher rates of undocumented cases among younger adults. Figure S2. Diagnostic yield of advanced premalignant lesions (APL) and colorectal cancer (CRC) by presenting symptom and age group (<50 vs. ≥50 years). Grouped bar chart showing the proportion (%) of colonoscopies detecting APL and CRC for each symptom category. Error bars represent 95% confidence intervals. Significant age-related differences (*p* < 0.05) were observed for rectal bleeding, abdominal pain, and bloody diarrhea, with higher yields in the ≥50 group. Color legend: gold (<50 years), blue (≥50 years). Figure S3. Age comparison of the number needed to scope (NNS) to detect one advanced colorectal neoplasia (ACRN) by symptom category. Bar plot showing NNS for patients <50 years (gold) and ≥50 years (blue) across major presenting symptoms. Lower bars indicate higher diagnostic efficiency. Loss of appetite and rectal bleeding had the lowest NNS values across both age groups, with older adults consistently demonstrating superior efficiency. Error bars represent 95% confidence intervals; asterisks (*) denote statistically significant between-group differences (*p* < 0.05).
