# Supplementary material for: Age-Specific Colonoscopic Yield and Symptom-Based Risk Stratification in Symptomatic Adults: A Bicenter Omani Analysis to Inform Early Detection and Screening Strategies for Colorectal Neoplasia
Source: Medicina (Kaunas). 2026 Feb 13;62(2):374. doi: 10.3390/medicina62020374 (PMC12942456; doi:10.3390/medicina62020374)
Supplement: Supplementary file 1 [file medicina-62-00374-s001.zip › medicina-4106550-supplementary.pdf]

Supplementary Materials:

Table S1. Symptom-specific diagnostic yield for advanced premalignant lesions (APL) and colorectal cancer (CRC), stratified by age group (<50 vs ≥50 years).

Symptom Category	Symptom	<50 yrs (N)	APL n (%)	CRC n (%)	≥50 yrs (N)	APL n (%)	CRC n (%)	APL p-value	CRC p-value
**GI Bleeding**	Rectal bleeding/hematochezia	174	9 (5.2%)	8 (4.6%)	221	13 (5.9%)	27 (12.2%)	0.933	**0.014**
	Bloody diarrhea	255	10 (3.9%)	9 (3.5%)	285	18 (6.3%)	29 (10.2%)	0.290	**0.004**
	Melena	32	2 (6.2%)	0 (0.0%)	96	10 (10.4%)	5 (5.2%)	0.729	0.330
**Bowel Habit Changes**	Chronic constipation	124	3 (2.4%)	4 (3.2%)	220	10 (4.5%)	18 (8.2%)	0.391	0.115
	Chronic diarrhea	105	4 (3.8%)	1 (1.0%)	76	5 (6.6%)	4 (5.3%)	0.495	0.163
	Altered bowel movements	56	0 (0.0%)	2 (3.6%)	51	0 (0.0%)	0 (0.0%)	1.000	0.496
**Abdominal Symptoms**	Abdominal pain/discomfort	202	11 (5.4%)	8 (4.0%)	199	11 (5.5%)	23 (11.6%)	1.000	**0.008**
	Abdominal distension/bloating	19	0 (0.0%)	2 (10.5%)	24	1 (4.2%)	1 (4.2%)	1.000	0.575

	Nausea	22	1 (4.5%)	1 (4.5%)	23	1 (4.3%)	6 (26.1%)	1.000	0.096
**Constitutional Symptoms**	Unintentional weight loss	51	1 (2.0%)	4 (7.8%)	77	5 (6.5%)	12 (15.6%)	0.401	0.306
	Loss of appetite	14	1 (7.1%)	2 (14.3%)	42	6 (14.3%)	9 (21.4%)	0.666	0.711
	Night sweats	3	0 (0.0%)	1 (33.3%)	3	0 (0.0%)	1 (33.3%)	1.000	1.000
**Other**	Anemia	63	2 (3.2%)	1 (1.6%)	248	22 (8.9%)	8 (3.2%)	0.186	0.692
	Anal symptoms	16	0 (0.0%)	0 (0.0%)	9	0 (0.0%)	1 (11.1%)	1.000	0.360

Values are expressed as n (%) of colonoscopies yielding APL or CRC within each symptom subgroup. p-values were obtained using the chi-square or Fisher's exact test, comparing age groups for each symptom category. Bolded p-values denote significance ($p < 0.05$). ***Abbreviations:*** *APL = advanced premalignant lesions; CRC = colorectal cancer; GI = gastrointestinal.*

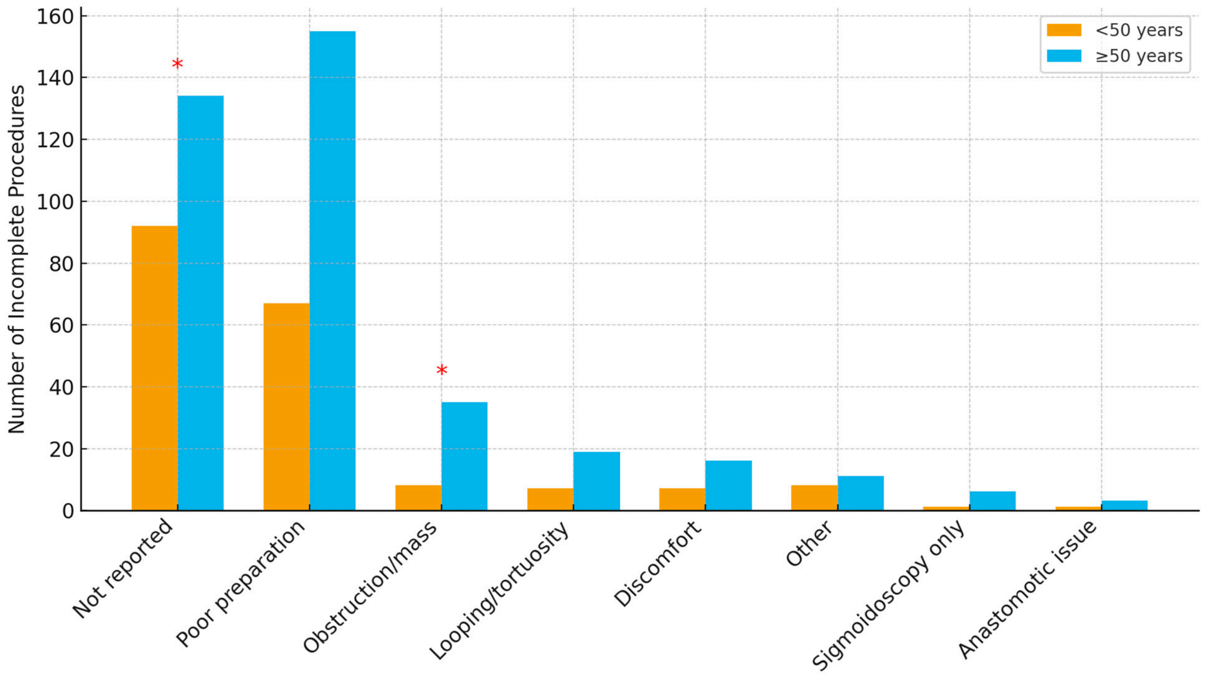

Figure S1. Distribution of reasons for incomplete colonoscopy by age group (<50 vs ≥50 years). Bars represent the number of incomplete procedures attributed to each cause. Asterisks (*) denote statistically significant differences between age groups ($p < 0.05$). The two most common causes were undocumented reasons and inadequate bowel preparation, with significantly higher rates of undocumented cases among younger adults.

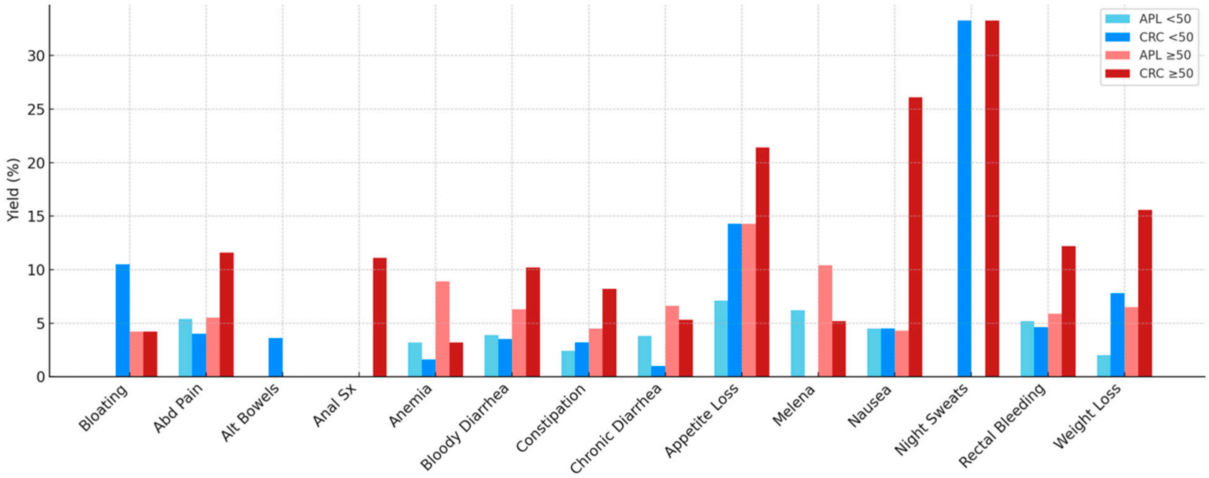

Figure S2. Diagnostic yield of advanced premalignant lesions (APL) and colorectal cancer (CRC) by presenting symptom and age group (<50 vs ≥50 years). Grouped bar chart showing the proportion (%) of colonoscopies detecting APL and CRC for each symptom category. Error bars represent 95 % confidence intervals. Significant age-related differences ($p < 0.05$) were observed for rectal bleeding, abdominal pain, and bloody diarrhea, with higher yields in the ≥50 group. Color legend: gold (<50 years), blue (≥50 years).

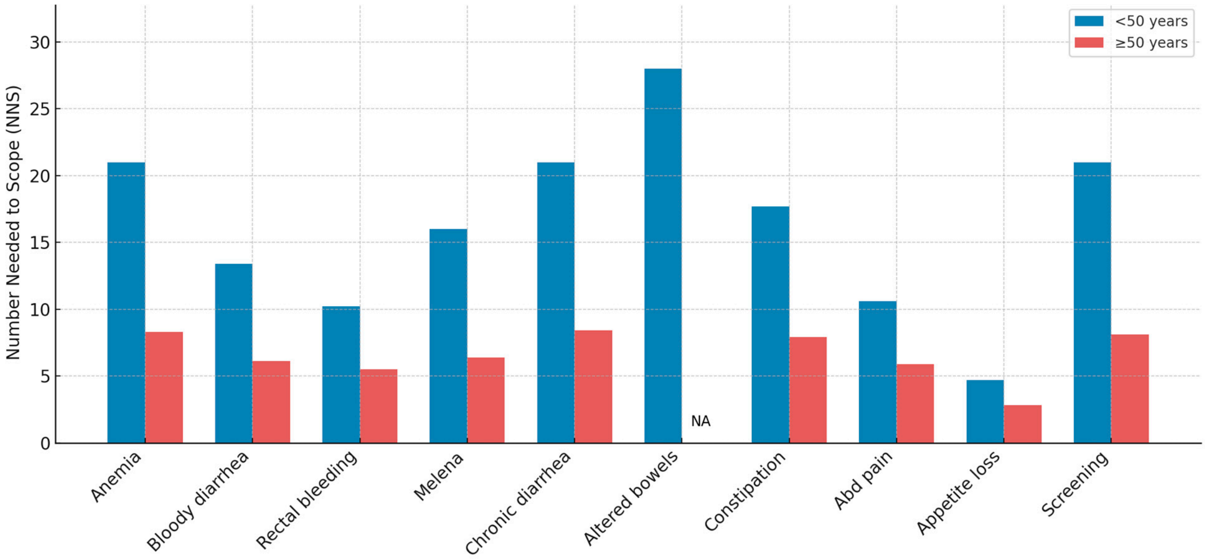

Figure S3. Age-stratified number needed to scope (NNS) to detect one advanced colorectal neoplasia (ACRN) by symptom category. Bar plot showing NNS for patients <50 years (gold) and ≥50 years (blue) across major presenting symptoms. Lower bars indicate higher diagnostic efficiency. *Loss of appetite* and *rectal bleeding* had the lowest NNS values across both age groups, with older adults consistently demonstrating superior efficiency. Error bars represent 95 % confidence intervals; asterisks (*) denote statistically significant between-group differences ($p < 0.05$).
